# Adaptation of a cold vapour mercury analyser to flow injection analysis

**DOI:** 10.1155/S1463924688000409

**Published:** 1988

**Authors:** Celio Pasquini, Wilson F. Jardim, Lourival C. de Faria

**Affiliations:** Instituto de Quimica – UNICAMP Universidade Estadual de Campinas – CP 6154 Campinas SP 13081 Brazil

## Abstract

Minor modifications to a Coleman MAS-50A Mercury Analyser System allowed the determination of mercury by flow injection analysis. Using sample volumes of 600 μl it was possible to analyse up to 120 samples per hour, with a detection limit of 0.2 μg. l^-1^ (120 pg) of mercury. The authors also report on a simple digestion procedure which replaces the time- and reagent-consuming EPA procedure, when the sample content permits.


Journal of Automatic Chemistry Vol. 10, No. 4 (October–December 1988), pp. 188–191

### Adaptation of a cold vapour mercury analyser to flow injection analysis

**Celio Pasquini, Wilson F. Jardim and Lourival C. de Faria**

*Instituto de Quimica – UNICAMP, Universidade Estadual de Campinas – CP 6154, 13081 Campinas, SP-Brazil*

*Minor modifications to a Coleman MAS-50A Mercury Analyser System allowed the determination of mercury by flow injection analysis. Using sample volumes of 600 μl it was possible to analyse up to 120 samples per hour, with a detection limit of 0·2 μg. $l^{-1}$ (120 pg) of mercury. The authors also report on a simple digestion procedure which replaces the time- and reagent-consuming EPA procedure, when the sample content permits.*

### Introduction

The determination of low concentrations of mercury is usually achieved by using the Cold Vapour Atomic Absorption (CVAA) technique [1]. Mercuric ions are reduced to elemental form usually employing $Sn^{+2}$ or $BH_4^-$ [2]. The metal is then swept from the solution with the help of a carrier gas (nitrogen, argon or air) to a detection cell, where the absorption of mercury is measured at 253·7 nm. A simple T-shaped open tube detection cell was used by Thompson and Thomerson [3] and Rooney [2].

Various commercially available instruments, such as the Coleman Mercury Analyser System MAS-50A and the Mercury Analyser from Buck Scientific, were developed using the CVAA principle [4 and 5]. However, their operation requires large amounts of both sample and time, as the absorption needs to be determined after reaching the steady state; furthermore, the manual operations and glassware usage increase the contamination risk.

A relatively small number of automated methods have been described in the literature which are based on the Segmented Continuous Flow approach, and these tend to suffer both poor analytical rate and high sample consumption [6–8].

A Flow Injection Analysis (FIA) system was developed [9] measuring the mercury diffused through a PTFE membrane after reduction employing $BH_4^-$. The system showed that the FIA approach is capable of combining a high rate of analysis with precision and low consumption of sample and reagents. However, further studies on the membrane characteristics will be necessary before it can be used as a routine methodology.

This paper describes an FIA system based on a new gas/liquid separation cell that can be used to improve the performance of classical commercial mercury analysers based on the CVAA principle. The system was easily implemented and a Coleman MAS-50A was used with only minor modifications.

A simplified digestion procedure is suggested in place of the time- and reagent-consuming EPA (Environmental Protection Agency, USA) method of pre-digestion for industrial effluents, generated in the Solvay process, used in the manufacture of sodium hydroxide and chlorine. This pre-digestion, which is used to convert organically-found mercury to the inorganic form, has been simplified [10]. However, its use does not seem to be necessary for Solvay effluents as the amount of organic mercury compounds present is negligible. The digestion is then mainly required to avoid the interference caused by sulphide used in the effluent treatment. Results obtained for mercury determinations using the EPA-recommended digestion and the proposed system are also compared.

### Experimental

### *Reagents*

All reagents were of analytical grade and deionized, doubly distilled water was used throughout. Glassware was cleaned overnight by soaking in 10% v/v nitric acid.

The 1000 mg. $l^{-1}$ mercury(II) stock solution was prepared by dissolving 0.1345 g of mercury(II) chloride in 100 ml of 10% v/v nitric acid.

### *Digestion procedures*

The following EPA digestion was used: 5 ml of concentrated sulphuric acid and 2·5 ml of concentrated nitric acid were added to 10·0 ml aliquots of sample. 15 ml of 5% w/v potassium permanganate solution was then added to this solution, followed, after 15 min, by 8 ml of 5% w/v of potassium persulphate solution. The sample was then heated for 2 h at 95 °C. After cooling the excess permanganate was destroyed by adding 6 ml of a 12% w/v hydroxylamine hydrochloride solution. Standard solutions and a blank used in the calibration procedure, were prepared by this same method.

The suggested simplified pre-digestion procedure was as follows. 1 ml of 1 : 1 v/v mixture of concentrated nitric acid and sulphuric acid was added to a 10·0 ml aliquot of sample. After cooling to room temperature, 0·6 ml of a 6% w/v of potassium permanganate solution was added. Sample determination was carried out within 15 min. Standard solutions and a blank were again prepared following this method.

### *Equipment*

A Coleman MAS-50A Mercury Analyser had its original

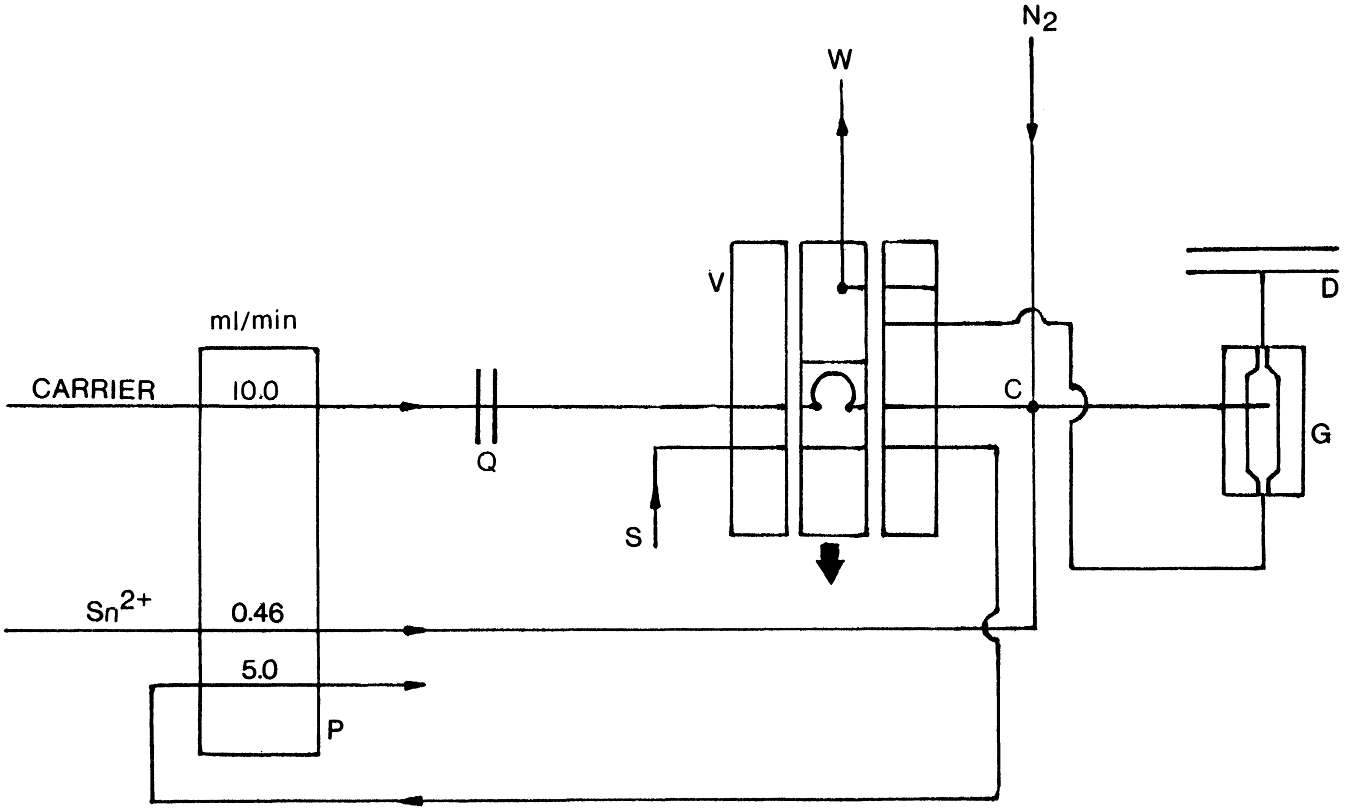

*Figure 1. FIA system schematic diagram. Where C = confluence point; D = detection cell; G = gas/liquid separation cell; P = peristaltic pump; Q = pulse damper; S = sample inlet; V = injection valve; W = to the water aspirator. Valve shown in injection position. See text for a more detailed description.*

closed detection cell replaced with an open Pyrex T-shaped cell, 150 mm long with an internal diameter of 7 mm, similar to that previously described [6]. An ECB-RE 101 chart recorder was used to collect the output signal from the MAS-50A.

A Micronal B-332 five-channel peristaltic pump fitted with Tygon pumping tubes was used for fluid flow control.

*FIA system*

The FIA manifold schematic diagram is shown in figure 1. Polyethylene tubing of 0·8 mm i.d. was used in the manifold construction, except where stated. A 35% w/v stannous chloride (QM – analytical grade CT 8697) in 1% v/v $HNO_3$ solution was used as the reducing agent when the simplified digestion procedure was followed, since part of the reductant was consumed by the excess permanganate. When using the EPA digestion, a 10% w/v stannous chloride in 1% v/v $HNO_3$ proved to be effective in reducing mercuric ions to their elemental form. A 1% v/v nitric acid solution was used as carrier. A flow rate of 200 ml. $min^{-1}$ of nitrogen (measured after the separation cell with the peristaltic pump off and at room temperature and pressure) was used to strip the elemental mercury out of the aqueous solution.

The gas/liquid separation cell is shown in figure 2. It was constructed using a 3 cm wide, 4·5 cm high and 2 cm thick acrylic block divided in two parts (A and B). Two 1 cm diameter holes leading to a 0·5 cm hole were drilled at the centres of both parts. Tygon tubes of 3·5 mm i.d. were glued at these 0·5 cm holes using cyanoacrylate glue. Part A contains, perpendicular to the cell body, a hole with a short glued Tygon tube and through which passed a 0·8 mm i.d. teflon tube which comes from the FIA manifold and reaches the centre of the cell. After construction, parts A and B were glued together so as to form a single unit. The upper aperture of the separation cell was connected to the T-shaped detection cell placed in the optical path of the CVVA using a 5 cm long Tygon tube. The bottom aperture was connected to the injection valve using a polyethylene tube.

Cyclic operation of the system described in figure 1 is started when the central part of the injection valve(V) is slid to the sampling position (moving in the direction shown by the arrow). While in this position, the sample loop (600 μl) is filled with the sample solution and the separation cell receives a gas/liquid mixture composed of the carrier, the $Sn^{+2}$ and nitrogen mixed at point C. This point is 3 cm from the injection valve. The water aspirator(W) is connected through the injection valve to the bottom aperture of the cell(G) aspirating liquid, nitrogen and some air through the detection cell(D). This enables both the separation and detection cells to be kept clean and leads to a flat base-line chart recorder being observed during this period.

When the central portion of the valve moves back (assuming the configuration shown in figure 1) the

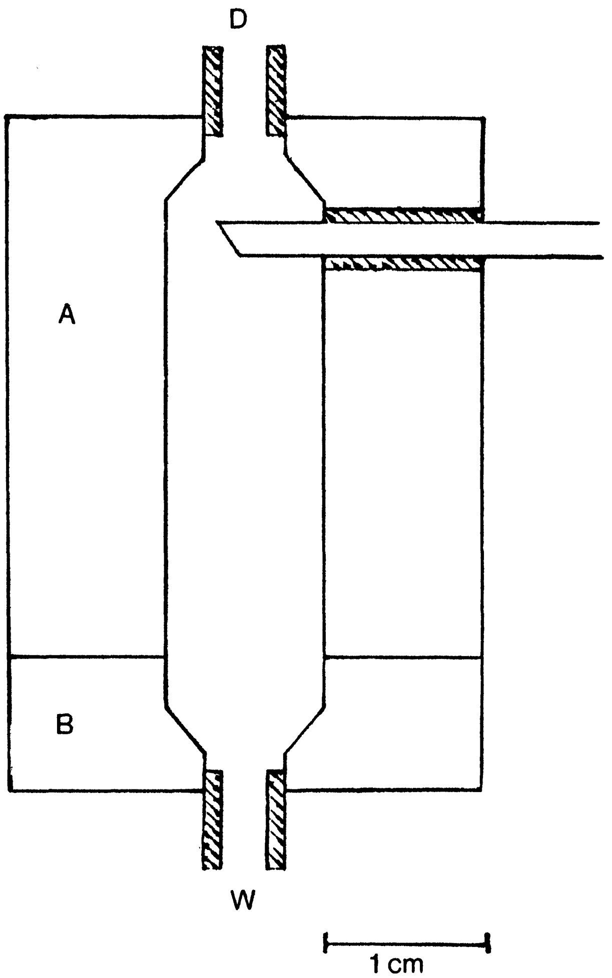

*Figure 2. Gas/liquid separation cell. Where A and B = acrylic parts; D = output to detection cell; W = output to water aspirator; I = inlet from FIA manifold.*

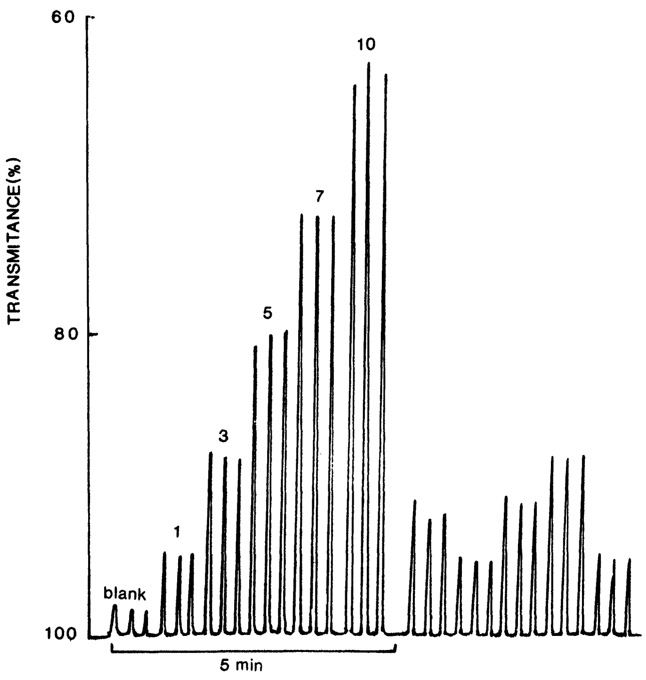

*Figure 3. Typical chart recorder output. A blank and five standard solutions followed by five samples. The numbers represents mercury concentration in µg. $l^{-1}$. All results obtained are triplicates.*

sample is carried to the confluence point(C) and then through a 40 cm polyethylene tube connected to the perpendicular inlet of the separation cell(G). The liquid starts to fill the cell(G) since the action of the aspirator is absent during this stage, the bottom aperture being virtually sealed. Simultaneously, the nitrogen containing the elemental mercury is directed to the detection cell(D) and a rising signal is observed on the chart recorder. As this signal level peaks, the level of the solution in the separation cell(G) has risen close to that of the inlet teflon tube. The operator, alerted by the falling signal, then replaces the valve in the sampling position, thus causing the solution and the mercury of the detection cell to be aspirated to waste. This operation cleans the system rapidly and during the sampling period the wall of the cell is continuously washed by the acidic carrier solution.

### Results and discussion

Optimization steps for the FIA system show that when other parameters are kept constant, increasing the sample volume leads to a steady state condition in which the signal peak height remains constant for large sample volumes. A equilibrium between the amount of mercury carried to the flow cell and that leaving it could explain this behaviour, as the sample volume necessary to achieve this situation is lower than the volume of the separation cell. Employing the conditions described in the experimental section of this paper, it was found that a sample volume of 600 µl is sufficient to allow a maximum peak height, whilst still allowing the signal to begin decreasing before the level of the liquid reaches the end of the separation cell.

It was also observed that the nitrogen flow rate is a critical parameter that demands special attention in order to maintain the reliability of the method. For a set of constant parameters, a lower nitrogen flow rate decreases the sensitivity. Increasing the flow rate leads to an increase in the signal peak height towards steady state. However, before this point can be achieved, the high flow rate begins carrying small droplets of solution to the detection cell. In the present system this causes an increase in the blank values and a lack of reproducibility because the elemental mercury retained in these drops is released during subsequent injections. This probably could be prevented by heating the detection flow cell above 100 °C, but this would require a more complex system than that described.

An important aspect of the proposed method is the fact that cleaning off the detection and separation cells is independent of the nitrogen flow rate used to strip out the elemental mercury from the solution. This, in fact, enables the nitrogen flow rate to be adjusted for maximum sensitivity, without restrictions usually related to the time required to purge the mercury present in the detection cell, furthermore, the suction provided by the water aspirator removes all mercury that was not carried to the detection cell, preventing sample carry-over.

*Table 1. Comparison between results for Solvay effluents.*

| Sample number | Mercury concentration (μg. $l^{-1}$)* EPA method | Proposed method |
|---|---|---|
| 1 | 2·2 | 2·2 |
| 2 | 1·6 | 1·6 |
| 3 | 1·6 | 1·7 |
| 4 | 1·2 | 1·5 |
| 5 | 1·8 | 2·0 |

* Mean of triplicate determinations.

A typical chart recorder output is shown in figure 3. The relationship between the mercury concentration and the signal peak height is linear to 25 μg. $l^{-1}$ of mercury. This range coincides with that usually found in effluents deriving from the Solvay process. In the range from 1 to 10 μg. $l^{-1}$ the precision, estimated as the mean relative standard deviation, was 4% ($N = 30$). The calculated detection limit [11] for the proposed procedure is 0·2 μg. $l^{-1}$ or 120 pg of mercury with the sample volume used. The sensitivity is 0·014 absorbance units. (l. $\mu g^{-1}$).

Solvay effluent samples containing 1·0 μg. $l^{-1}$ of mercury show an average recovery of 96% when spiked up to 3·0 μg. $l^{-1}$. A comparison beween the EPA digestion and the proposed digestion procedure applied to Solvay effluents is presented in table 1 and shows a satisfactory agreement between the two sets of data. However, it is worth mentioning that in this particular type of industrial effluent, the amount of possible organic interferents is negligible. The effect of the presence of sulphide up to 50 mg. $l^{-1}$ was overcome by the simplified digestion procedure and the interference caused by chloride up to 10 000 mg. $l^{-1}$ was negligible if the analysis is performed within 20 min of potassium permanganate addition.

### Acknowledgement

The authors are grateful to Dr P. Moss for revising the manuscript.

### References

1. Hatch, R. and Ott, W. L., *Analytical Chemistry*, **40** (1968), 2085.
2. Rooney, R. C. *Analyst*, **101** (1976), 678.
3. Thompson, K. C. and Thomerson, D. R., *Analyst*, **99** (1974), 595.
4. Dumarey, R., Heindrychz, R., Dams, R. and Hoste, J. *Analytica Chimica Acta*, **107** (1979), 159.
5. Kivalo, P., Visapda, A. and Bachman, R., *Analytical Chemistry*, **46** (1974), 1814.
6. Goto, M., Shibakawa, T., Arita, T. and Ishu, D., *Analytica Chimica Acta*, **140** (1982), 179.
7. Oda, C. E. and Ingle, J. O., Jr., *Analytical Chemistry*, **53** (1981), 2030.
8. Satueman, B. T., *Applied Spectroscopy*, **39** (1985), 48.
9. Andrade, J. C., Pasquini, C., Baccan, N. and Van Loon, J. C., *Spectrochimica Acta*, **38B** (1983), 1329.
10. Fray, B. J., Nelson, L. A., and Rolph, M. G., *Analyst*, **103** (1978), 656.
11. *Nomenclature, Symbols, Units and their Usage in Spectrochemical Analysis – II. Data Interpretation. Spectrochimica Acta*, **33B** (1978), 245.

### CHEMICAL SENSORS CLUB NEWS

The latest issue of *CSC News* (No. 10) concentrates on the implications of the UK's imminent Control of Substances Hazardous to Health (COSHH) Regulations. The legislation, which should come into effect during 1989, will be of particular interest to those involved in sensor development and application. It seems likely that the UK industry will be involved in a significantly higher level of monitoring, particularly of levels of atmospheric contaminants, both in the air and in biological fluids.

The role that sensors could play, the type of measurements that will be required and the practical limitations on the size and weight of devices are all outlined in the *CSC News* article.

In effect, a future market is being defined, including a list of analytes, their concentrations of interest, and device requirements. Industrial hygienists will be seeking means to evaluate worker exposure and will be ready to take advantage of any available sensors.

The features in the newsletter are book reviews and a variety of new items.

*Copies of 'CSC News' are available from the Editor, 87 Gower Street, London WC1E 6AA. Details about joining the Chemical Sensors Club from Ric Treble, Laboratory of the Government Chemist, Cornwall House, Waterloo Road, London SE1 8XY.*

